# Primary ovarian insufficiency associated with pazopanib therapy in a breast angiosarcoma patient: A CARE-compliant case report

**DOI:** 10.1097/MD.0000000000018089

**Published:** 2019-12-16

**Authors:** Rita De Sanctis, Elena Lorenzi, Elisa Agostinetto, Tania D’Amico, Matteo Simonelli, Armando Santoro

**Affiliations:** a Department of Medical Oncology and Hematology, Humanitas Cancer Center, Humanitas Clinical and Research Center – IRCCS, Rozzano (MI); b Humanitas University, Department of Biomedical Sciences, Pieve Emanuele – Milan; c Department of Experimental Medicine, Endocrinology-Pituitary Disease, “Sapienza” University of Rome, Rome, Italy.

**Keywords:** amenorrhea, breast angiosarcoma, gonadal toxicity, ovarian insufficiency, pazopanib

## Abstract

**Rational:**

The growing population of young cancer survivors and a trend toward postponing pregnancy until later years in life are leading to a deeper attention towards understanding treatment-induced sequelae, and, in particular, the effects of cancer and/or treatment on fertility. Nowadays, the infertility risks potentially associated with molecular targeted therapies are not established, and clinical reports are sparse. Moreover, the increasing use of molecular targeted drugs in the adjuvant setting and in diseases with better prognosis makes preservation of fertility a major topic in current research.

**Patient's concerns:**

Here, we report the case of an 18-year-old woman, with a 3-cm superficial lump of the right breast, who had no remarkable family or medical history. Menarche had occurred at the age of 14 years, with normal regular periods.

**Diagnosis:**

High-grade angiosarcoma, with metastatic progression and multiple relapse, was diagnosed.

**Interventions:**

After diagnosis, right radical mastectomy was carried out with no evidence of residual disease. No adjuvant treatment was delivered. Lymph node metastasis were found later and chemotherapy with doxorubicin 25 mg/m^2^/day and ifosfamide 1 g/m^2^/day (both on days 1–3) every 21 days was administered. During treatment, the patient reported menstrual irregularities but no amenorrhea. Due to further local relapse a few years later, the patient was treated for progressive metastatic disease with gemcitabine 1000 mg/m^2^ on days 1 and 8 every 21 days for 6 cycles, and underwent surgery, followed by pegylated liposomal doxorubicin, 50 mg/m^2^ on day 1 every 28 days. After further disease progression 5 years after first diagnosis, pazopanib was administered at a dose of 800 mg daily for 10 months.

**Outcomes:**

The patient experienced a transient ovarian insufficiency possibly due to pazopanib. Since amenorrhea developed within 2 months from the initiation of pazopanib treatment and menses returned regularly only after discontinuation of the treatment itself.

**Lessons:**

This is the first case report that strongly suggests a correlation between pazopanib exposure and development of ovarian insufficiency. Our case tantalizes to inspire additional preclinical and clinical research on the true incidence, possible dose dependence, and reversibility of pazopanib (and other TKIs) -induced ovarian failure.

## 1 Introduction

In recent decades, the number of cancer patients in western countries has dramatically increased; two-thirds of them are expected to survive at least 5 years from diagnosis.^[1]^ In total, 5% of cancer patients are diagnosed before the age of 40 years.^[2]^ Many cancer survivors must cope with long-lasting effects of their disease and treatments. For those with reproductive potential, treatment-related infertility is one of the most relevant consequences leading to serious psychological distress, which in turn leads to a negative impact on the quality of life.

Angiosarcomas are rare vascular neoplasms accounting for approximately 2% of all adult soft tissue sarcomas (STS), with an aggressive clinical behavior and a very poor prognosis. Of note, primary angiosarcomas of the breast are most commonly diagnosed in patients aged 20 to 40 years, when the gonadal toxicity is a major concern.^[3]^ The 5-year overall survival (OS) rate for non-metastatic cases is 30% to 40%,^[4]^ and local recurrence rates are up to 70%.^[5]^ A complete surgical resection with wide margins remains to be the treatment backbone. Adjuvant radiotherapy should maximize local control,^[6]^ but no impact on OS has been demonstrated.^[7]^ The use of adjuvant or neoadjuvant chemotherapy is still controversial.^[8,9]^ In the metastatic setting, median OS is 8 months. Anthracycline-based regimens represent the standard first-line therapy,^[10]^ while paclitaxel^[11]^ and gemcitabine^[12]^ have shown some activity with a typical median progression-free survival (PFS) of 4 months. Inhibition of angiogenesis is another relevant therapeutic strategy in STS. Pazopanib has been demonstrated to significantly increase median PFS from 1.6 to 4.6 months vs placebo in advanced STS, progressing after a first-line chemotherapy. Encouraging data on sorafenib in vascular sarcomas have also been published (6-month PFS of 31%–35%).^[13]^ Of note, the sample size of angiovascular sarcoma subgroup was quite small in clinical trials on STS, so conclusive results on different agents are hard to define.

Pazopanib is an oral multitargeted tyrosine kinase inhibitor (TKI) that acts against vascular endothelial growth factor receptors (VEGFRs) −1, −2, and −3, and platelet-derived growth factor receptors (PDGFRs) −α, and −β and c-kit,^[14]^ which has been approved for the treatment of advanced renal cell carcinoma^[15]^ and non-adipocytic STS.^[16]^ Ovarian failure is not a recognized complication of treatment with pazopanib. Here, we report the case of a young woman with metastatic angiosarcoma of the breast who developed a transient ovarian insufficiency during treatment with pazopanib.

## 2 Case report

An 18-year-old woman, with a 3-cm superficial lump of the right breast, underwent a surgical excision in January 2011 at another Institution, with a diagnosis of high-grade angiosarcoma. She had no remarkable family or medical history. Menarche had occurred at age of 14 years, with normal regular periods. In February 2011, she was referred to the Humanitas Research Hospital, Milan, for further work-up. After staging procedures, a right radical mastectomy was carried out with no evidence of residual disease at the pathological examination. No adjuvant treatment was delivered.

In October 2011, a positron emission tomography/computed tomography (PET/CT) scan revealed a pathologic uptake in the right axilla and the patient underwent an axillary dissection. The pathological examination was consistent with lymph node metastasis of high-grade angiosarcoma. From November to December 2011, adjuvant chemotherapy with doxorubicin 25 mg/m^2^/day and ifosfamide 1 g/m^2^/day (both on days 1–3) every 21 days was administered. During treatment, the patient reported menstrual irregularities but no amenorrhea.

In January 2013, a nodule of the right thoracic wall was surgically removed confirming a relapse of angiosarcoma. Adjuvant radiation therapy was proposed, but the patient refused it.

In September 2013, a PET/CT scan revealed a further local relapse along with the involvement of the contralateral axillary lymph nodes for which our patient underwent chemotherapy with gemcitabine 1000 mg/m^2^ on days 1, 8 every 21 days for 6 cycles, achieving a partial response according to RECIST criteria, followed by surgery.

In May 2016, for progressive disease with distant pleuropulmonary metastases and further local recurrence, the patient received pegylated liposomal doxorubicin 50 mg/m^2^ on day 1 every 28 days, with stable disease after 3 cycles and disease progression after 6 cycles. Therefore, in November 2016, pazopanib at a dose of 800 mg daily was started. Thyroid function tests at the baseline were within the normal range (thyroid-stimulating hormone [TSH] 2.25 mIU/l with normal range of 0.27–4.20). In December 2016, thyroid monitoring showed an increase in TSH (22.83 mIU/l), a decrease in fT3 1.8 pg/ml (normal range: 2.0–4.4) and normal fT4 10.6 pg/ml (normal range: 9.3–17.1). Anti-thyroglobulin and anti-thyroid peroxidase antibodies were absent. A thyroid ultrasound excluded the presence of nodules or other abnormalities. Levothyroxine replacement therapy was started at a dose of 50 μg daily. In January 2017, secondary amenorrhea occurred. Beta-HCG dosage was negative (2.5 mIU/ml, n.r. 0–5.3). In February 2017, thyroid tests showed a stable TSH level (22.87 mIU/l), and a normalization of fT3 (2.5 pg/ml). Dose of levothyroxine was increased to 75 μg daily. A PET/CT scan showed a complete response of the lung lesions and a partial response of the local relapse. In March 2017, while still on therapy with pazopanib, normal thyroid function was restored (TSH 4.21 mIU/l, fT3 3 pg/ml, fT4 11.9 pg/ml) and reproductive hormone levels were as follows: follicle-stimulating hormone (FSH) was 48 mIU/ml, luteinizing hormone (LH) was 75.8 mIU/ml, and estradiol (E2) was 61 pg/ml. Anti-Müllerian hormone (AMH) was 3.27 ng/ml (within the lower values of the normal range: normal range: 0.96–13.34 for 18–25-year-old women, 0.03–3.27 for 41–45-year-old women). Antral follicle count (AFC) obtained by trans-vaginal ultrasound revealed a right AFC of 4 and a left AFC of 2 (normal count: 7).^[17]^ Based on these data, we diagnosed a primary ovarian insufficiency. In September 2017, a PET/CT scan showed a local progression of disease, pazopanib was stopped and a new treatment with paclitaxel was proposed. After discussion with the patient about possible treatment-related adverse events, patient's refusal of hair-loss inducing therapies and clinical benefit from gemcitabine previously administered, a further line of treatment with gemcitabine was started in October 2017. In November 2017, after 2 months from pazopanib discontinuation, menses returned regularly. Hormonal assays showed FSH 9.2 mIU/ml, LH 10.6 mIU/ml, E2 120 pg/ml, in the follicular phase.

In April 2018, the patient experienced further disease progression and paclitaxel-based treatment has been proposed. The patient has provided a written informed consent for the case details to be published; approval from the ethical committee was not necessary, in line with the regulations of our Institution.

## 3 Discussion

To the best of our knowledge this is the first case report that strongly suggests a correlation between pazopanib exposure and development of ovarian insufficiency, since amenorrhea developed within 2 months from the initiation of pazopanib treatment and menses returned regularly only after discontinuation of the treatment itself.

Pazopanib has recently been approved for the treatment of patients with advanced renal cell carcinoma as first-line treatment or after cytokines and metastatic non-adipocytic STS after previous chemotherapy (typically anthracycline-based, taxanes, or gemcitabine). The most common pazopanib-associated toxicities were diarrhea, fatigue, nausea, hypertension, decreased appetite, dysgeusia, hypothyroidism and palmar-plantar erythrodysesthesia syndrome.^[15,16,18]^ Gonadal toxicity has not been reported as a possible side effect of treatment with pazobanib in any clinical trial so far.

Despite its high selectivity, this molecule also affects signal transduction in normal cells and tissue, causing a wide range of previously unknown on- and off-target side effects. The hypothesis of a primary ovarian insufficiency linked to pazopanib treatment is plausible, since kinase signaling pathways targeted by this drug play a crucial role in the formation, maturation, and survival of oocytes and follicles. Indeed, c-Kit and PDGFR are expressed both in oocytes and granulosa cells, respectively, and during ovarian development, their activation promotes the transition from primordial to primary follicle.^[19]^ Furthermore, VEGF is a key regulator of angiogenesis that is recognized to play a crucial role in the development of corpus luteum, a highly vascularized, endocrine organ supporting pregnancy development.^[20]^ In murine models, injection of the neutralizing anti-VEGFR-2 antibody disrupts function of corpus luteus during pregnancy, as evidenced by reduced organ size, regression of luteal vessels, and decreased progesterone secretion.^[19]^ Moreover, inhibition of VEGFR-2 caused removal of endothelial cells from the vascular basement membrane and apoptosis of luteal steroid-producing epithelial cells. The effect of antibody was shown to be specific to the ovary and administration of an antibody against VE-cadherin (E4G10), which specifically blocks endothelial proliferation, did not disrupt luteal function, thus supporting the notion that VEGFR-2-mediated endothelial cell signals are critical to maintain functionality of luteal blood vessels. In a pre-clinical study, female fertility was affected by treatment with pazopanib, likely due to the suppressive effect of this drug on the endometriotic tissue expressions of VEGF and CD117.^[21]^ A decreased number of corpora lutea along with an increased number of ovarian cyst and ovarian atrophy were observed in rats, even if without significant changes in the ovarian follicle number and ovarian reserve.^[22]^

In the clinical management of our patient, we first ruled out the most frequent causes of amenorrhea by performing a pregnancy test and an accurate global endocrine assessment. A rapid onset hypothyroidism was observed only few weeks after the pazopanib initiation; however, the thyroid function returned to normal level in a few months after the introduction of replacement therapy. Anyhow, our patient continued to be amenorrhoeic until pazopanib discontinuation, despite the normalization of thyroid function.

Most of the available literature on infertility risk of antineoplastic agents reports rates of amenorrhea, although this is an imprecise surrogate measure of infertility. Nowadays, we have more precise and reliable methods to assess chemotherapy-induced gonadal toxicity (FSH, LH, estradiol levels) and fertility potential (AMH and AFC).^[17]^ Aside from the timing of amenorrhea onset, the strength of the hypothesized correlation relies also on the variation of fertility markers during treatment. At the onset of amenorrhea, beta-HCG was dosed to exclude a potential pregnancy; we did not dose FSH and LH since amenorrhea could be related to hypothyroidism. After 3 months of treatment, when thyroid function was normalized but menses did not restore, we noticed high FSH and LH levels with low estradiol ones. The AFC was dramatically decreased with a low AMH level comparable to the one of 40-year-old women. Considering these findings, in our case we can hypothesize a hypergonadotropic hypogonadism which is compatible with a direct ovarian damage. Nevertheless, the normalization of hormone values after treatment discontinuation suggests that the damage caused by pazopanib could be transient.

In the absence of literature data, this case report points out the necessity to better define the impact on female fertility of pazopanib, as well as other antiangiogenetic drugs, such as sunitinib, sorafenib, and bevacizumab. Currently, mainly pre-clinical studies are reported in the literature.^[23]^ The largest animal study (rats) investigating the effects of sunitinib on female fertility did not reveal treatment-related effects on fertility.^[21]^ On the contrary, the study demonstrated that sorafenib affected ovarian function in rats by delaying the development of ovaries and inducing central necrosis of the corpora lutea. The effects of bevacizumab on fertility have been specifically discussed in a recent review.^[24]^

For cancer patients with reproductive potential, treatment-related infertility is one of the most relevant issue leading to psychological distress and a negative impact on quality of life. The young age of onset of breast angiosarcomas underlines the importance of an appropriate counselling about possible treatments’ impact on fertility. Further data are required in order to increase awareness about this topic and to address therapeutic choices, especially when dealing with young patients in fertile age. Current ASCO guidelines require assessment of the infertility risk for every antineoplastic agent before starting treatment, along with an accurate communication with the patient and eventual referral to fertility preservation specialists.^[25]^ This issue is even more complicated if we consider the metastatic setting where the referral to fertility preservation strategies can be considered as controversial because of the poor prognosis of the patient. However, the use of antiangiogenetic drugs and, more in general of targeted therapies, is increasingly growing in advanced diseases, thus resulting in long-term clinical benefit and disease control (e.g., anti-VEGFR and anti-EGFR in colorectal cancer, EGFR TKIs, and ALK inhibitors in lung cancer). In our case, in contrast to literature data on prognosis of metastatic angiosarcomas, we observed a longer than expected OS. Based on this, despite the different life expectancies, the oncologist should be able to offer an appropriate counseling before starting every antineoplastic treatment. On the other hand, fertility preservation should be taken into account in adjuvant patients, who could be currently treated with targeted therapies, such as imatinib (an anti-ABL, c-Kit and PDGFR TKI) in high-risk GIST,^[26]^ and – according to latest Food and Drug Administration approval – sunitinib (a multi-TKI) in high-risk renal cell carcinoma,^[27]^ dabrafenib (a selective mutant-BRAF inhibitor) and trametinib (a MEK inhibitor) in stage III V600E/K-mutant melanoma.^[28]^ Data on long-term gonadal toxicities from adjuvant exposure to TKIs are lacking. In the near future, the increasing use of these drugs both in adjuvant and metastatic setting will be a challenge for oncologists and fertility specialists treating patients with no evidence of disease and a moderate-to-high probability of surviving their disease.

Our case tantalizes to inspire additional preclinical and clinical research on the true incidence, possible dose dependence, and reversibility of pazopanib (and other TKIs) -induced ovarian failure.

## Acknowledgments

Editorial assistance was provided by Luca Giacomelli, PhD, and Aashni Shah, of Polistudium srl, whose assistance was supported by internal funds.

## Author contributions

**Conceptualization:** Rita De Sanctis, Armando Santoro.

**Investigation:** Rita De Sanctis, Elena Lorenzi, Elisa Agostinetto, Tania D’Amico, Matteo Simonelli, Armando Santoro.

**Supervision:** Armando Santoro.

**Writing – original draft:** Rita De Sanctis.

**Writing – review & editing:** Rita De Sanctis, Elena Lorenzi, Elisa Agostinetto, Tania D’Amico, Matteo Simonelli, Armando Santoro.
